# Concurrent radiotherapy with gefitinib in elderly patients with esophageal squamous cell carcinoma: Preliminary results of a phase II study

**DOI:** 10.18632/oncotarget.5193

**Published:** 2015-09-10

**Authors:** Yaping Xu, Yuanda Zheng, Xiaojiang Sun, Xinmin Yu, Jialei Gu, Wei Wu, Gu Zhang, Jinlin Hu, Wenyong Sun, Weimin Mao

**Affiliations:** ^1^ School of Medicine, Shandong University, Jinan, China; ^2^ Department of Radiation Oncology, Zhejiang Cancer Hospital, Hangzhou, China; ^3^ Department of Medical Oncology, Zhejiang Cancer Hospital, Hangzhou, China; ^4^ Department of Pathology, Zhejiang Cancer Hospital, Hangzhou, China; ^5^ Zhejiang Key Laboratory of the Diagnosis & Treatment Technology on Thoracic Oncology, Hangzhou, China

**Keywords:** esophageal cancer, elderly patients, epidermal growth factor receptor, gefitinib, radiotherapy

## Abstract

The survival rate associated with esophageal cancer is very poor due to diagnosis at advanced stages of disease and insensitivity to chemotherapy. This study investigated the efficacy of gefitinib combination with radiation in 20 elderly patients with esophageal squamous cell carcinoma (ESCC) who were not eligible for platinum-based chemotherapy. Immunohistochemistry was performed to analyze epidermal growth factor receptor (EGFR) expression, and the amplified refractory mutation system was used to detect EGFR mutations. Treatment response was assessed by endoscopy and computed tomography. Treatment toxicity was evaluated using the National Cancer Institute's Common Toxicity Criteria. The data showed that among these 20 patients, 5 experienced a complete response (CR), 13 a partial response (PR), and 2 had stable disease. The overall response rate (CR + PR) was 90%, the median overall survival (OS) was 14.0 months (95% confidence interval [CI]: 10.0–17.9 months), and the median progression-free survival was 7.0 months (95% CI: 0–17.2 months). Patients with good Eastern Cooperative Oncology Group performance status, never smoking, and EGFR mutated tumors had the best OS (14.0, 14.0, and 17.0 months, respectively). Treatment-related grade 3/4 toxicity occurred in five patients. No case of grade 3/4 impaired liver function or hematological toxicity was observed. Concurrent radiotherapy with gefitinib is effective and tolerable in elderly ESCC patients.

## INTRODUCTION

Esophageal cancer is a significant worldwide health problem that accounted for one-sixth of cancer-related deaths in 2008 in the world. [1] Histologically, esophageal cancer is classified into esophageal squamous cell carcinoma (ESCC) and adenocarcinoma. ESCC is the major histological type of esophageal cancer in the world and is one of the most aggressive malignancies, whereas esophageal adenocarcinoma frequently occurs in Western countries, such as the USA. [2] Furthermore, approximately 20% of esophageal cancers are diagnosed in patients over 75 years of age. [3] Although the past three decades have seen improvements in perioperative care and surgical techniques, the introduction of multimodal treatment, and the feasibility of surgery for the majority of esophageal cancer patients, treatment of elderly patients still represents a challenge for surgeons, and surgery-related mortality in elderly patients undergoing esophagectomy is as high as 18%. [4] Thus, chemoradiotherapy is more frequently used in esophageal cancer patients, which is mainly based on an early randomized trial that compared radiotherapy (64 Gy) with four courses of concurrent cisplatin, 5-flurouracil (5-FU), and radiotherapy (50.4 Gy) and showed a significant survival benefit for patients. [5] However, because geriatric patients are traditionally excluded from randomized controlled clinical trials for a variety of reasons (heterogeneity, comorbidities, inability to consent, etc.), chemotherapy for elderly esophageal cancer patients, especially patients with advanced age, together with coexisting severe medical morbidities, is not always tolerable. [6] Thus, the search for novel concurrent or target therapies for elderly esophageal cancer patients is urgently needed.

Furthermore, the epidermal growth factor receptor (EGFR) signaling pathway plays an important role in the regulation of homeostasis, such as cell growth, differentiation, and carcinogenesis. [7–10] Altered EGFR expression or EGFR mutation has been reported in esophageal cancer and correlated with poor patient prognosis and inferior response to therapy. [8–11] Moreover, radiation can induce autophosphorylation of EGFR protein and downstream substrates, which then leads to tumor resistance to radiotherapy, and tyrosine kinase inhibitors can prevent this autophosphorylation of EGFR. Thus, drugs that target EGFR (cetuximab, erlotinib, and gefitinib) in combination with radiotherapy have been proven to be effective for treating certain solid tumors, such as head and neck cancer, [12, 13] non-small cell lung cancer, [14] and rectal cancer [15] with a favorable toxicity profile. Gefitinib (also called ZD1839 or Iressa; AstraZeneca, Wilmington, DE) is a specific tyrosine kinase inhibitor of EGFR. Previous studies showed that gefitinib treatment of non-small cell lung cancer patients with EGFR exon 19 and 21 mutations achieves a dramatic clinical response. [16, 17] Gefitinib is well tolerated at a dose of 250 mg/day, with skin rash and diarrhea as the main toxicities. [18] In esophageal cancer, we found three studies using EGFR tyrosine kinase inhibitors plus thoracic radiation to treat ESCC patients. [19–21] In addition, detection of EGFR mutations is a useful and sensitive biomarker to predict the effect of an EGFR tyrosine kinase inhibitor on non-small cell lung cancer. [22] Therefore, in the present study, we conducted a phase II clinical trial in elderly Chinese ESCC patients to evaluate concomitant gefitinib and thoracic radiotherapy in terms of both feasibility and efficacy for the treatment of ESCC and to study the impact of EGFR alterations on patient survival.

## RESULTS

### Patient characteristics

This study included 20 elderly ESCC patients who were recruited from Zhejiang Cancer Hospital between September 2010 and November 2012. The patient characteristics are listed in Table 1. Briefly, there were 13 males and 7 females with a mean age of 76 years (range, 65–83 years). Of these 20 patients, there were 12 TNM stage III/IV, and all 20 patients were ineligible for and not treated with platinum-based chemotherapy, due to neuropathy (*n* = 2), cardiac disease (*n* = 7), poor performance status (*n* = 2), or poor overall health (*n* = 9).

**Table 1:** Baseline characteristics of the patients

Clinical characteristics	No. of patients (%)
All Patients	20 (100%)
Gender	
male	13 (65)
female	7 (35)
Age (years)	
Median (range)	76 (65–83)
ECOG PS	
0 and 1	18 (90)
2	2 (10)
TNM stage (UICC 2002)	
II : T_2–3_N_0_M_0_, T_1–2_N_1_M_0_	8 (40)
III : T_3_N_1_M_0_, T_4_N_any_M_0_	10 (50)
IV: T_any_N_any_M_1a_	2 (10)
Cigarettes/year	
≥400	8 (40)
Never smoked	12 (60)
*EGFR expression	
High	8 (40)
Low	7 (35)
N/A	5 (25)
*EGFR mutation	
Positive	3 (15)
Negative	12 (60)
N/A	5 (25)

ECOG PS, Eastern Cooperative Oncology Group performance status; TNM, tumor-node-metastasis; EGFR, epidermal growth factor receptor, N/A, not available because of insufficient tissues.

### Treatment response and survival of patients

Among the 20 included patients, 18 (90%) received the full dose of radiotherapy (50.4 Gy at 1.8 Gy/fraction), whereas two patients (10%) received a lower dose of radiotherapy (45.0 and 48.6 Gy) due to grade 3 esophagitis. However, one patient did not receive the second month of gefitinib due to a swallowing problem and esophagitis during the radiotherapy course. The treatment response of each patient was assessed by esophagography, CT scans, and endoscopy performed between 4 weeks after completion of this concurrent treatment and documented using RECIST. As shown in Table 2, 5 cases of CR, 13 cases of PR, and two cases of stable disease (SD) were observed among these 20 patients after concurrent radiotherapy with gefitinib. The overall response rate (CR + PR) was 90%, which satisfied the pre-defined goal of an end point response rate (CR plus PR) of more than 85%.

**Table 2:** Treatment efficacy of the patients

Efficacy	No. of patients (%)
Response	
Overall response rate	18 (90%)
Disease control rate	
Complete response	5 (25%)
Partial response	13 (65%)
Stable disease	2 (10%)
Progressive disease	0 (0)
Survival	
Median (months)	14.0 (95% CI: 10.0–17.9)
1 year (%)	58.2
Progression-free survival	
Median (months)	7.0 (95% CI: 0–17.2)

**Figure 1:**
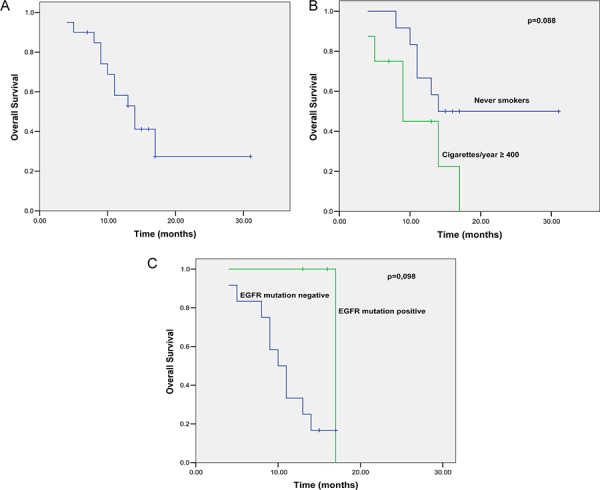
Kaplan-Meier curves for OS **A.** Kaplan-Meier curves for OS. **B.** Kaplan-Meier curves for OS stratified by tobacco smoking status (log-rank test: *p* = 0.088). **C.** Kaplan-Meier curves for OS stratified by EGFR mutation status (log-rank test: *p* = 0.098).

The median follow-up period was 17 months (range, 6–31 months). During the follow-up, 12 (60%) patients died, and 13 (65%) patients experienced disease progression. The remaining 7 (35%) patients were free of disease progression. The median OS of these patients was 14.0 months (95% confidence interval [CI]: 10.0–17.9 months; Figure 1A). The associations of the clinicopathological features of these patients with OS are summarized in Table 3. In particular, a better OS rate occurred in patients with good ECOG performance status (14 vs. 4 months, *p* = 0.000), and the OS was marginally better among patients who had never smoked (14 vs. 9 months; *p* = 0.088) or those with a mutated EGFR tumor (10 vs. 17 months, *p* = 0.098; Figure 1B, 1C).

**Table 3:** Association of clinicopathological data with OS of the patients

Clinicopathological data	Median overall survival (months)	*p*
All Patients	14	
Gender		
Male	14	0.873
Female	13	
ECOG PS		
0,1	14	0.000
2	4	
TNM stage (UICC 2002)		
I/II	14	0.795
III/IV	11	
Cigarettes/year		
≥400	9	0.088
Never smoked	14	
*EGFR expression		
High	10	0.736
Low	13	
*EGFR mutation		
Positive	17	0.098
Negative	10	

ECOG PS, Eastern Cooperative Oncology Group performance status; TNM, tumor-node-metastasis; EGFR, epidermal growth factor receptor.

* EGFR expression and mutation status were evaluated in 15 patients (5 patients had insufficient material).

### Treatment toxicity and safety issues

Acute adverse effects are summarized in Table 4. The addition of gefitinib to thoracic radiation therapy was generally well tolerated, and the most common toxicities were esophagitis (95%) and tracheitis (55%). Grade 3 esophagitis only developed in four patients (20%), although grade 1 or higher toxicities occurred in approximately 50% patients, including pneumonitis, vomiting, fatigue, and rash. The most noticeable adverse effects were grade 1/2 and were well controlled by supportive care. There was no grade 3/4 impaired liver function or hematological toxicity observed in these patients.

**Table 4:** Acute toxicities after treatment

Grade	0	1	2	3	Total (1 + 2 + 3)
Esophagitis	5%(1)	20%(4)	55%(11)	20%(4)	95%
Tracheitis	45%(9)	40%(8)	15%(3)	0	55%
Pneumonitis	55%(11)	25%(5)	15%(3)	5%(1)	45%
Fatigue	50%(10)	30%(6)	15%(3)	5%(1)	50%
Vomiting	50%(10)	35%(7)	15%(3)	0	50%
Rash	60%(12)	15%(3)	25%(5)	0	40%
Diarrhea	65%(13)	25%(5)	10%(2)	0	35%
Leucopenia	80%(16)	15%(3)	5%(1)	0	20%
Hemoglobin	85%(17)	10%(2)	5%(1)	0	15%
Platelet count	95%(19)	0	5%(1)	0	5%
Weight loss	90%(18)	10%(2)	0	0	10%

### Treatment failure

By 18 months after treatment, 13 patients had experienced relapse and/or distant metastases. The first site of relapse was a primary tumor lesion in 7 patients (53.8%), distant metastasis in 5 patients (38.4%), intrapulmonary metastasis in 2 patients (15.3%), bone metastasis in 1 patient (7.7%), and distant lymph node metastasis in 1 patient (7.7%). Only one patient experienced recurrence of disease simultaneously in the primary tumor lesion and at a distant site in the liver. Furthermore, 12 patients died during the follow-up period, and the causes of the death included progression of a primary tumor in four patients (33.3%), bacterial pneumonia in 4 patients (33.3%), tracheoesophageal fistula in 1 patient (8.3%), and an unspecified cause in 3 patients (25.0%).

### Expression of EGFR protein and *EGFR* mutations

**Figure 2:**
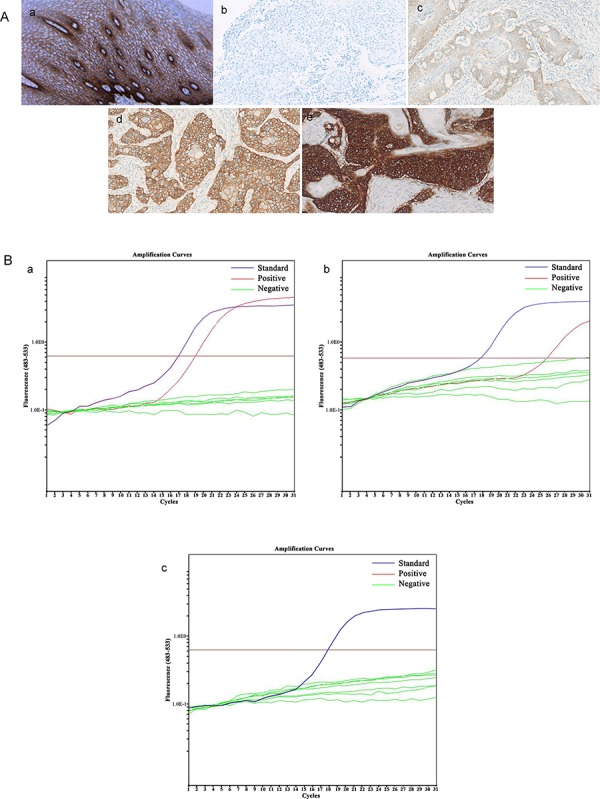
Alteration of EGFR expression and EGFR mutation **A.** Expression of EGFR protein: in normal esophageal epithelium (a); negative, no discernible staining or background type staining (b); 1+, definite cytoplasmic staining and/or equivocal discontinuous membrane staining (c); 2+, unequivocal membrane staining with moderate intensity (d); and 3+, strong and complete plasma membrane staining (e). **B.** *EGFR* mutation: mutated EGFR (a-b); wild-type EGFR (c).

EGFR protein expression and *EGFR* mutations were analyzed in 15 patients (5 patient had insufficient tissue material). Immunohistochemical staining showed that two patients had no discernible EGFR expression; five patients showed 1+ expression of EGFR in tumors; five patients showed 2+ expression of EGFR in tumors; and three had ESCC with a 4+ level of EGFR expression (Figure 2A). After treatment, patients with ESCC expressing high levels (2+ and 3+ expression) of EGFR had a median OS of 13 months compared to 10 months in patients with an ESCC tumor showing a low level of EGFR expression, although this difference was not statistically significant (*p* = 0.537; Table 3).

Furthermore, EGFR was mutated in three patients (20%), whereas the rest of the 12 patients did not have EGFR exon 19–21 mutations (Figure 2B). After the treatment, patients with a tumor exhibiting EGFR mutation had a median OS of 17 months compared to 10 months in patients whose tumors were without an EGFR mutation (*p* = 0.098; Table 3 and Figure 1C).

## DISCUSSION

The results of our current study suggest that concomitant treatment with gefitinib and thoracic radiotherapy in elderly ESCC patients had significant clinical value. The overall response rate (CR + PR) was 90%, which exceeded the goal per our study design. Only one patient did not receive the second month gefitinib due to side effects of radiotherapy (esophagitis and swallowing problem). The median OS of our patients was 14.0 months (95% CI, 10.0–17.9 months), which was better than the OS of 9.3 months with radiotherapy alone reported in the RTOG-8501 trial [23]. Furthermore, our current study is the first such trial conducted in China for documenting the feasibility and efficacy of the treatment.

For this clinical trial, we recruited 20 ESCC patients, and the results showed a median OS of 14.0 months and a 1-year survival rate of 58.2%. For comparison, in the RTOG-8501 trial, [23] the 1-year survival rate was 34% with radiation and 52% with platinum-based chemoradiation therapy. The low numbers of patient responses to these treatments may because only 59% of the patients thought to be candidates for chemoradiation regimen actually completed the planned chemoradiation therapy. Moreover, only one other previous study assessed the combination of an EGFR tyrosine kinase inhibitor (erlotinib) with thoracic radiation therapy for treating ESCC patients in Asia. [21] Similar to the results of our current study, that study reported a median OS and progression-free survival (PFS) of 21.1 and 12 months, respectively. The 2-year OS, PFS, and local-regional relapse-free survival rates were 44.4%, 38.9%, and 66.7%, respectively. Thus, gefitininb combined with radiation therapy is a better choice for elderly ESCC patients who are ineligible for platinum-based chemotherapy.

Furthermore, the current study was the first to correlate tobacco smoking and other clinicopathological data with treatment responses. We found that those who had never smoked had a better treatment response to gefitinib/radiation therapy than did heavy smokers, although this difference was not statistically significant. Tobacco smoking could indeed lead to a low response to another EGFR tyrosine kinase inhibitor, erlotinib, due to 25% lower bioavailability and an increase in its major metabolite OSI-420 (due to faster clearance). [24] Moreover, we only obtained 15 of 20 prospectively banked tissues samples from the patients for detection of EGFR alterations. Based on previous studies of non-small cell lung cancer, *EGFR* mutations occurred more common in non-smoking patients. [25–28] EGFR gene mutation status has not been prospectively studied in esophageal cancer, although it has been shown to significantly correlate with the response of lung cancer patients to EGFR-TKI therapy. [29, 30] In our current study, despite the small number of patients limiting the statistical power to draw a definite conclusion, it is intriguing that an EGFR mutation was associated with a better outcome tendency in gefitinib-treated patients (OS, 17 vs. 10 months in ARMS-positive and -negative patients, respectively). In addition, patients with ESCC expressing high levels of EGFR had a better OS than patients with an ESCC tumor with low EGFR expression, although this difference was not statistically significant. However, a previous phase II study of gefitinib as a second-line treatment for advanced esophageal cancer reported a significantly higher disease control rate (response and SD) in patients with ESCC expressing high levels of EGFR. [31] However, to date, the use of an EGFR alteration (overexpression or mutation) as a predictive marker for treatment efficacy is still controversial; thus, the design of a proper multidisciplinary clinical trial will confirm the usefulness of EGFR alteration as a biomarker for EGFR-targeted therapy of ESCC patients. In addition, our current study also showed that concurrent radiotherapy with gefitinib is effective in elderly patients with ESCC without EGFR overexpression or mutation, indicating that gefitinib could help to sensitize tumor cells to the effect of radiotherapy. However, further study is needed to clarify whether and how gefitinib can be used to sensitize the effect of radiotherapy on tumor cells.

In addition, the present study also showed that this regimen of therapy was generally well-tolerated by the patients. The addition of daily gefitinib to thoracic radiation therapy was associated with only mild toxicity. Most adverse effects were grade I/II and easily controlled by supportive care. For patients with grade III esophagitis, treatment was delayed for about 1 week. Only one patient developed grade III pneumonitis at 2 months after concurrent treatment, and there were no cases of grade 3/4 impaired liver function or hematological toxicity observed in these patients. Thus, the data from the current study support the safety of concomitant treatment with gefitinib and thoracic radiotherapy in ESCC patients.

However, our current study does have some limitations. For example, no true control group was included in this a single-arm, phase II clinical trial, and only ESCC patients were recruited in this study. The incidence of esophageal adenocarcinoma is dramatically increasing in Western countries. Therefore, the results of this study may be not generalized to apply for North American and European patients. Secondly, although daily oral 250 mg gefitinib was administered for 2 months concurrently to the radiotherapy, the optimal duration was not determined, and further studies are needed to identify the optimal targeting treatment duration for esophageal cancer. Thirdly, the incidence of EGFR mutations is different with various sensitivity detection methods. [32] In the current study, we detected an EGFR mutation with ARMS, which is a highly sensitive method, and the incidence of EGFR mutations in our patients was relatively higher than that reported previously. [7, 9, 10] Finally, although the data presented herein appears promising, this study is relatively small, and more data from randomized trials are needed to further validate this regimen.

In conclusion, our current data suggest that concomitant treatment with gefitinib and thoracic radiotherapy was well tolerated and effective in elderly ESCC patients. Our study revealed better outcomes in patients with good ECOG performance status, a history of not smoking, and EGFR mutation; however, we did not compare clinical outcomes, including survival rate, local disease control, distant metastasis, and treatment response, directly with those by common treatment modalities, such as chemoradiotherapy. Therefore, a future randomized study is needed to further confirm the benefits of concomitant treatment with gefitinib and thoracic radiotherapy in ESCC patients.

## MATERIALS AND METHODS

### Patient eligibility

In this study, we recruited 20 elderly (65 years or older) patients with biopsy-proven primary ESCC who were ineligible for and not treated with platinum-based chemotherapy, due to neuropathy (*n* = 2), cardiac disease (*n* = 7), poor performance status (*n* = 2), or poor overall health (*n* = 9). All patients had an Eastern Cooperative Oncology Group (ECOG) performance status of 0–2. The clinical stage was determined by a computed tomography (CT) scan of the chest and abdomen, barium-swallow X-ray, and endoscopic ultrasonography. If necessary, positron emission tomography-computed tomography (PET-CT) scan was performed. TNM stage IVa patients were included, but only if a distant metastasis occurred in celiac or cervical lymph nodes, but not in other sites. Leukocyte counts were ≥3,000/mm^3^, the absolute neutrophils ≥1,500/mm^3^ and platelets ≥100,000/mm^3^. Serum levels of bilirubin, aspartate transaminase (AST), and alanine transaminase (ALT) were within the normal range, and alkaline phosphatase was ≤2.5 times the institutional upper limit. Bronchoscopy with biopsy and cytology was performed if the trachea or bronchus was involved. The exclusion criteria were as follows: patients with concurrent malignancies other than basal cell/squamous cell skin cancer, *in situ* cervical cancer, or transitional cell bladder cancer; a history of allergy to gefitinib or its excipients; life expectancy <3 months; prior anti-EGFR therapy; lack of physical integrity of the gastrointestinal tract or malabsorption that would impair the absorption of the study drug; and clinically active interstitial lung disease, or concomitant use of CYP 3A4/5 inducers or inhibitors.

The current study was approved by the Ethical Review Committee of Zhejiang Cancer Hospital (Hangzhou, China). Recommendations of the Declaration of Helsinki for biomedical research involving human subjects were also followed. Each patient signed a written informed consent before participating in this study, and a copy of the written consent is available for review upon request.

### Study design, treatment, and assessment

The study was designed as a single center, non-random, phase II study of open-label gefitinib with radiotherapy as a definite treatment for esophageal cancer in elderly patients. Gefitinib was administered at a daily oral dose of 250 mg for 2 months without interruption until it was stopped due to excessive toxicity, disease progression, or patient request. In cases of excessive toxicity, interruptions were allowed up to 14 days. If the adverse effect did not return to below grade 2, dose reduction by 250 mg after 2 days was allowed, but no more than one dose reduction was permitted. Toxicity was assessed according to the National Cancer Institute Common Toxicity Criteria grading system. External beam radiotherapy was delivered on day 1 using high-energy linear accelerators with a intensity modulated radiation therapy technique and CT simulation to define the gross tumor volume. The locoregional draining lymph nodes were included in the clinical target volume. Patients received 1.8 Gy/fraction for a total dose of 50.4 Gy. The radiation fields extended 3.5 cm beyond the proximal and distal extent of the primary tumor lesions, and the lateral borders were 1.0–1.5 cm beyond tumor lesions. The dosage was prescribed to the target volume. Paraffin-embedded tumor tissue blocks were collected from the initial diagnostic biopsies of the patients. Complete blood counts, including differential counts, as well as a complete metabolic panel, including liver and kidney function tests, were assessed weekly during the treatment. Treatment response was assessed by esophagography, CT scans, and endoscopy that were performed 4 weeks after completion of radiotherapy and analyzed according to the RECIST (Response Evaluation Criteria in Solid Tumors). The treatment toxicity was evaluated using the National Cancer Institute's Common Toxicity Criteria (CTCAE 4.0) and recorded according to the worst score achieved during treatment. All data evaluations and assessments were conducted in a blinded fashion.

### Immunohistochemistry

Immunohistochemistry (IHC) was performed on formalin-fixed, paraffin-embedded tissue sections of tissues from eligible ESCC cases. Consecutive tissue sections were used to verify the histologic staging. For IHC, the sections were first deparaffinized in xylene and then subjected to antigen retrieval in 10 mM citrate buffer (pH 9.0) with microwave irradiation and up to 3% hydrogen peroxide treatment. After that, the sections were incubated with a primary pre-diluted mouse anti-EGFR antibody (clone #2-18C9; DAKO, Hamburg, Germany), a monoclonal mouse anti-human Ki67 antibody (clone #MIB-1; DAKO; at 1:400 dilution), or a monoclonal mouse anti-human cyclin-D1 antibody (cat #RM-9104-S; Neomarker, Fremont, CA, USA; at 1:25) at 4°C overnight. On the next day, the sections were further incubated with an EnVision kit indirect peroxidase system (DAKO) and visualized using 3,3′-diaminobenzidine (DAB) as a chromogen. The sections were then counterstained with hematoxylin and viewed under a microscope to assess the percentage and intensity of nuclear and non-nuclear staining in tumor cells as well as background staining by two independent observers in a blinded manner.

The intensity of immunostaining was scored using a four-tier system: negative, no discernible staining or background type staining; 1+, definite cytoplasmic staining and/or equivocal discontinuous membrane staining; 2+, unequivocal membrane staining with moderate intensity; and 3+, strong and complete plasma membrane staining. Samples exhibiting 2+ or 3+ immunostaining are classified as highly expressing EGFR.

### Detection of *EGFR* mutations

*EGFR* mutations at exons 19–21 were detected using a Therascreen RGQ PCR kit (Qiagen, Hilden, Germany), which uses the Scorpions technology and the amplified refractory mutation system (ARMS) to detect *EGFR* mutations after real-time PCR amplification. This sensitive method can detect 29 types of *EGFR* mutations, and the experiments were performed according to the manufacturer's instructions as described previously. [33]

### Statistical analysis

The number of patients required was estimated according to the methodology by Makuch and Simon for a phase II clinical trial [34]. This clinical trial was designed as a prospective, single center, non-random, phase II study. The primary end point of the study was the response rate, and the secondary end points were survival time and toxicity with this concurrent radiotherapy/gefitinib regimen. The study was designed to measure a response rate (cases of CR plus those of PR) of 85% compared with a minimal, clinically meaningful response rate of 70%. Upon employing an α = 0.05 and a β = 0.20, the target number of patients required to achieve this level of significance was 20 cases.

Given the known risks of concomitant therapy, a toxicity analysis was planned after 10 patients had completed treatment. Toxicity was judged unacceptable if four or more of these 10 patients had at least grade III esophageal or pulmonary toxicity.

PFS of the patients was measured from the beginning of treatment until disease progression, and OS was calculated from the first day of initiation of treatment until death or until the last follow-up examination. The survival curves were calculated using the Kaplan-Meier method. Associations between patient characteristics and survival were assessed using the log-rank test. The considered variables included: gender, ECOG score, TNM stage, tobacco smoke, and EGFR status. The data were analyzed anonymously. Statistical analysis was performed using SPSS software, Version 19.0 (SPSS Inc., Chicago, IL, USA). All probability values were two-sided, and *p* value <0.05 was considered statistically significant.

## References

[1] Pisani P, Parkin DM, Ferlay J (1993). Estimates of the worldwide mortality from eighteen major cancers in 1985. Implications for prevention and projections of future burden. Int J Cancer.

[2] Zhang J, Dhakal IB, Zhao Z, Li L (2012). Trends in mortality from cancers of the breast, colon, prostate, esophagus, and stomach in East Asia: role of nutrition transition. Eur J Cancer Prev.

[3] Nallapareddy S, Wilding GE, Yang G, Iyer R, Javle M (2005). Chemoradiation is a tolerable therapy for older adults with esophageal cancer. Anticancer Res.

[4] Poon RT, Law SY, Chu KM, Branicki FJ, Wong J (1998). Esophagectomy for carcinoma of the esophagus in the elderly: results of current surgical management. Ann Surg.

[5] al-Sarraf M, Martz K, Herskovic A, Leichman L, Brindle JS, Vaitkevicius VK, Cooper J, Byhardt R, Davis L, Emami B (1997). Progress report of combined chemoradiotherapy versus radiotherapy alone in patients with esophageal cancer: an intergroup study. Journal of clinical oncology : official journal of the American Society of Clinical Oncology.

[6] Kumagai K, Rouvelas I, Tsai JA, Mariosa D, Klevebro F, Lindblad M, Ye W, Lundell L, Nilsson M (2014). Meta-analysis of postoperative morbidity and perioperative mortality in patients receiving neoadjuvant chemotherapy or chemoradiotherapy for resectable oesophageal and gastro-oesophageal junctional cancers. Br J Surg.

[7] Yamada T, Alpers DH, Laine L, Owyang C, Powell DW (1999). Cancer Epidemiology.

[8] Montesano R, Hollstein M, Hainaut P (1996). Genetic alterations in esophageal cancer and their relevance to etiology and pathogenesis: a review. Int J Cancer.

[9] Hanawa M, Suzuki S, Dobashi Y, Yamane T, Kono K, Enomoto N, Ooi A (2006). EGFR protein overexpression and gene amplification in squamous cell carcinomas of the esophagus. Int J Cancer.

[10] Kitagawa Y, Ueda M, Ando N, Ozawa S, Shimizu N, Kitajima M (1996). Further evidence for prognostic significance of epidermal growth factor receptor gene amplification in patients with esophageal squamous cell carcinoma. Clin Cancer Res.

[11] Gotoh M, Takiuchi H, Kawabe S, Ohta S, Kii T, Kuwakado S, Katsu K (2007). Epidermal growth factor receptor is a possible predictor of sensitivity to chemoradiotherapy in the primary lesion of esophageal squamous cell carcinoma. Japanese journal of clinical oncology.

[12] Bonner JA, Harari PM, Giralt J, Cohen RB, Jones CU, Sur RK, Raben D, Baselga J, Spencer SA, Zhu J, Youssoufian H, Rowinsky EK, Ang KK (2010). Radiotherapy plus cetuximab for locoregionally advanced head and neck cancer: 5-year survival data from a phase 3 randomised trial, and relation between cetuximab-induced rash and survival. Lancet Oncol.

[13] Rao K, Kalapurakal S, Chalasani P, Robinson K, Malone J, Clausen C, Ronen O, Dhiwakar M, Shevlin B, Robbins KT (2013). A phase II study of intra-arterial cisplatin with concurrent radiation and erlotinib for locally advanced head and neck cancer. Cancer Chemother Pharmacol.

[14] Center B, Petty WJ, Ayala D, Hinson WH, Lovato J, Capellari J, Oaks T, Miller AA, Blackstock AW (2010). A phase I study of gefitinib with concurrent dose-escalated weekly docetaxel and conformal three-dimensional thoracic radiation followed by consolidative docetaxel and maintenance gefitinib for patients with stage III non-small cell lung cancer. J Thorac Oncol.

[15] Blaszkowsky LS, Ryan DP, Szymonifka J, Borger DR, Zhu AX, Clark JW, Kwak EL, Mamon HJ, Allen JN, Vasudev E, Shellito PC, Cusack JC, Berger DL, Hong TS (2014). Phase I/II study of neoadjuvant bevacizumab, erlotinib and 5-fluorouracil with concurrent external beam radiation therapy in locally advanced rectal cancer. Ann Oncol.

[16] Lynch TJ, Bell DW, Sordella R, Gurubhagavatula S, Okimoto RA, Brannigan BW, Harris PL, Haserlat SM, Supko JG, Haluska FG, Louis DN, Christiani DC, Settleman J, Haber DA (2004). Activating mutations in the epidermal growth factor receptor underlying responsiveness of non-small-cell lung cancer to gefitinib. The New England journal of medicine.

[17] Paez JG, Janne PA, Lee JC, Tracy S, Greulich H, Gabriel S, Herman P, Kaye FJ, Lindeman N, Boggon TJ, Naoki K, Sasaki H, Fujii Y, Eck MJ, Sellers WR, Johnson BE (2004). EGFR mutations in lung cancer: correlation with clinical response to gefitinib therapy. Science (New York, NY).

[18] Wolf M, Swaisland H, Averbuch S (2004). Development of the novel biologically targeted anticancer agent gefitinib: determining the optimum dose for clinical efficacy. Clin Cancer Res.

[19] Iyer R, Chhatrala R, Shefter T, Yang G, Malhotra U, Tan W, Levea C, Robins M, Khushalani N (2013). Erlotinib and radiation therapy for elderly patients with esophageal cancer - clinical and correlative results from a prospective multicenter phase 2 trial. Oncology.

[20] Zhang XB, Xie CY, Li WF, Zhang P, Wu SX (2012). [Phase II study of radiotherapy plus erlotinib for elder patients with esophageal carcinoma]. Zhonghua yi xue za zhi.

[21] Zhai Y, Hui Z, Wang J, Zou S, Liang J, Wang X, Lv J, Chen B, Zhu H, Wang L (2013). Concurrent erlotinib and radiotherapy for chemoradiotherapy-intolerant esophageal squamous cell carcinoma patients: results of a pilot study. Dis Esophagus.

[22] Abedi-Ardekani B, Dar NA, Mir MM, Zargar SA, Lone MM, Martel-Planche G, Villar S, Mounawar M, Saidi F, Malekzadeh R, Hainaut P (2012). Epidermal growth factor receptor (EGFR) mutations and expression in squamous cell carcinoma of the esophagus in central Asia. BMC Cancer.

[23] Cooper JS, Guo MD, Herskovic A, Macdonald JS, Martenson JA, Al-Sarraf M, Byhardt R, Russell AH, Beitler JJ, Spencer S, Asbell SO, Graham MV, Leichman LL (1999). Chemoradiotherapy of locally advanced esophageal cancer: long-term follow-up of a prospective randomized trial (RTOG 85-01). Radiation Therapy Oncology Group. JAMA.

[24] Li X, Kamenecka TM, Cameron MD (2009). Bioactivation of the epidermal growth factor receptor inhibitor gefitinib: implications for pulmonary and hepatic toxicities. Chem Res Toxicol.

[25] Sun YH, Fang R, Gao B, Han XK, Zhang JH, Pao W, Chen HQ, Ji HB (2010). Comparable rate of EGFR kinase domain mutation in lung adenocarcinomas from Chinese male and female never-smokers. Acta Pharmacol Sin.

[26] Kim HR, Shim HS, Chung JH, Lee YJ, Hong YK, Rha SY, Kim SH, Ha SJ, Kim SK, Chung KY, Soo R, Kim JH, Cho BC (2012). Distinct clinical features and outcomes in never-smokers with nonsmall cell lung cancer who harbor EGFR or KRAS mutations or ALK rearrangement. Cancer.

[27] D'Angelo SP, Pietanza MC, Johnson ML, Riely GJ, Miller VA, Sima CS, Zakowski MF, Rusch VW, Ladanyi M, Kris MG (2011). Incidence of EGFR exon 19 deletions and L858R in tumor specimens from men and cigarette smokers with lung adenocarcinomas. Journal of clinical oncology : official journal of the American Society of Clinical Oncology.

[28] Sholl LM, Yeap BY, Iafrate AJ, Holmes-Tisch AJ, Chou YP, Wu MT, Goan YG, Su L, Benedettini E, Yu J, Loda M, Janne PA, Christiani DC, Chirieac LR (2009). Lung adenocarcinoma with EGFR amplification has distinct clinicopathologic and molecular features in never-smokers. Cancer Res.

[29] Chen X, Zhu Q, Liu Y, Liu P, Yin Y, Guo R, Lu K, Gu Y, Liu L, Wang J, Wang Z, Roe OD, Shu Y, Zhu L (2014). Icotinib is an active treatment of non-small-cell lung cancer: a retrospective study. PloS one.

[30] Jeon JH, Kang CH, Kim HS, Seong YW, Park IK, Kim YT (2015). Prognostic and predictive role of epidermal growth factor receptor mutation in recurrent pulmonary adenocarcinoma after curative resection. European journal of cardio-thoracic surgery : official journal of the European Association for Cardio-thoracic Surgery.

[31] Janmaat ML, Gallegos-Ruiz MI, Rodriguez JA, Meijer GA, Vervenne WL, Richel DJ, Van Groeningen C, Giaccone G (2006). Predictive factors for outcome in a phase II study of gefitinib in second-line treatment of advanced esophageal cancer patients. Journal of clinical oncology : official journal of the American Society of Clinical Oncology.

[32] Cui Y, Chang D, Liu M, Lin C, Zhao B, Zhang X, Gong M (2013). Identification of exon 19 and 21 mutations of EGFR gene in Chinese patients with esophageal squamous cell carcinoma. World J Surg Oncol.

[33] Kimura H, Kasahara K, Kawaishi M, Kunitoh H, Tamura T, Holloway B, Nishio K (2006). Detection of epidermal growth factor receptor mutations in serum as a predictor of the response to gefitinib in patients with non-small-cell lung cancer. Clin Cancer Res.

[34] Makuch RW, Simon RM (1980). Sample size considerations for non-randomized comparative studies. J Chronic Dis.

